# Solubility-Weighted Index: fast and accurate prediction of protein solubility

**DOI:** 10.1093/bioinformatics/btaa578

**Published:** 2020-06-19

**Authors:** Bikash K Bhandari, Paul P Gardner, Chun Shen Lim

**Affiliations:** Department of Biochemistry, School of Biomedical Sciences, University of Otago, Dunedin, New Zealand; Department of Biochemistry, School of Biomedical Sciences, University of Otago, Dunedin, New Zealand; Biomolecular Interaction Centre, University of Canterbury, Christchurch, New Zealand; Department of Biochemistry, School of Biomedical Sciences, University of Otago, Dunedin, New Zealand

## Abstract

**Motivation:**

Recombinant protein production is a widely used technique in the biotechnology and biomedical industries, yet only a quarter of target proteins are soluble and can therefore be purified.

**Results:**

We have discovered that global structural flexibility, which can be modeled by normalized *B*-factors, accurately predicts the solubility of 12 216 recombinant proteins expressed in *Escherichia coli*. We have optimized these *B*-factors, and derived a new set of values for solubility scoring that further improves prediction accuracy. We call this new predictor the ‘Solubility-Weighted Index’ (SWI). Importantly, SWI outperforms many existing protein solubility prediction tools. Furthermore, we have developed ‘SoDoPE’ (Soluble Domain for Protein Expression), a web interface that allows users to choose a protein region of interest for predicting and maximizing both protein expression and solubility.

**Availability and implementation:**

The SoDoPE web server and source code are freely available at https://tisigner.com/sodope and https://github.com/Gardner-BinfLab/TISIGNER-ReactJS, respectively. The code and data for reproducing our analysis can be found at https://github.com/Gardner-BinfLab/SoDoPE_paper_2020.

**Supplementary information:**

Supplementary data are available at *Bioinformatics* online.

## 1 Introduction

High levels of protein expression and solubility are two major requirements of successful recombinant protein production (Esposito and Chatterjee, 2006). However, recombinant protein production is a challenging process. Almost half of recombinant proteins fail to be expressed and half of the successfully expressed proteins are insoluble (http://targetdb.rcsb.org/metrics/). These failures hamper protein research, with particular implications for structural, functional and pharmaceutical studies that require soluble and concentrated protein solutions (Hou *et al.*, 2018; Kramer *et al.*, 2012). Therefore, solubility prediction and protein engineering for enhanced solubility is an active area of research. Notable protein engineering approaches include mutagenesis, truncation (i.e. expression of partial protein sequences) or fusion with a solubility-enhancing tag (Chan *et al.*, 2010; Costa *et al.*, 2014; Esposito and Chatterjee, 2006; Kramer *et al.*, 2012; Trevino *et al.*, 2007; Waldo, 2003).

Protein solubility, in part, depends upon extrinsic factors such as ionic strength, temperature and pH, as well as intrinsic factors—the physicochemical properties of the protein sequence and structure, including molecular weight, amino acid composition, hydrophobicity, aromaticity, isoelectric point, structural propensities and the polarity of surface residues (Chiti *et al.*, 2003; Diaz *et al.*, 2010; Tartaglia *et al.*, 2004; Wilkinson and Harrison, 1991). Many solubility prediction tools have been developed around these features using statistical models (e.g. linear and logistic regression) or other machine learning models (e.g. support vector machines and neural networks) (Habibi *et al.*, 2014; Hebditch *et al.*, 2017; Heckmann *et al.*, 2018; Hirose and Noguchi, 2013; Sormanni *et al.*, 2017; Wu *et al.*, 2019; Yang *et al.*, 2019).

In this study, we investigated the experimental outcomes of 12 216 recombinant proteins expressed in *Escherichia coli* from the ‘Protein Structure Initiative: Biology’ (PSI: Biology) (Acton *et al.*, 2005; Chen *et al.*, 2004). We showed that protein structural flexibility is more accurate than other protein sequence properties in solubility prediction (Craveur *et al.*, 2015; Vihinen *et al.*, 1994). Flexibility is a standard feature appears to have been overlooked in previous solubility prediction attempts. On this basis, we derived a set of 20 values for the standard amino acid residues and used them to predict solubility. We call this new predictor the ‘Solubility-Weighted Index’ (SWI). SWI is a powerful predictor of solubility, and a good proxy for global structural flexibility. In addition, SWI outperforms many existing *de novo* protein solubility prediction tools.

## 2 Materials and methods

### 2.1 Data

We retrieved 12 216 PSI: Biology entries from the DNASU database (Acton *et al.*, 2005; Chen *et al.*, 2004; Seiler *et al.*, 2014). These proteins were previously expressed in *E.coli* using pET21_NESG or pET15_NESG expression vectors (*N* = 8780 and 3436, respectively). For validation, we used the solubility data of *E.coli* proteins from eSOL (*N* = 3198; http://www.tanpaku.org/tp-esol/index.php?lang=en) (Niwa *et al.*, 2009). See also Supplementary Figure S1 and Table S1A.

In addition, we downloaded the ‘stickiness’ data of 397 *E.coli* proteins to examine the effects of surface amino acid residues (http://www.weizmann.ac.il/Structural_Biology/faculty_pages/ELevyintDef/interface_def.html) (Levy *et al.*, 2012).

### 2.2 Protein sequence properties

The standard protein sequence properties were calculated using the Bio.SeqUtils.ProtParam module of Biopython v1.73 (Cock *et al.*, 2009). All miscellaneous protein sequence properties were computed using the R package protr v1.6-2 (Xiao *et al.*, 2015).

### 2.3 Protein solubility prediction

We used the standard and miscellaneous protein sequence properties to predict the solubility of the PSI: Biology and eSOL targets. For method comparison, we chose the protein solubility prediction tools that are scalable (Table 1). Default configurations were used for running the command line tools.

**Table:** Table 1. Comparison of protein solubility prediction methods and software

	Approaches	Features	Wall time (s per sequence)^a^	PSI: Biology (AUC)^b^	eSOL [*R_s_* (*P*-value)]
SWI	Arithmetic mean (this study). Sequence composition scoring using a set of 20 values for amino acid residues derived from Smith *et al.*’s normalized *B*-factors. Trained and tested using the PSI: Biology dataset curated by DNASU (Seiler *et al.*, 2014). Available at https://tisigner.com/sodope and https://github.com/Gardner-BinfLab/SoDoPE_paper_2020.	1	**0.00 ± 0.00**	**0.71 ± 0.01**	0.50 (2.51 × 10^−205^)
Protein-Sol	Linear model (Hebditch *et al.*, 2017). Trained and tested using eSOL dataset (Niwa *et al.*, 2009). Available at https://protein-sol.manchester.ac.uk/.	10	1.16 ± 0.75	0.68 ± 0.02	**0.54 (2.37 × 10^−240^)**
Flexibility	A sliding window of nine amino acid residues using Vihinen *et al.*’s normalized *B*-factors. Available at https://github.com/biopython/biopython.	1	0.38 ± 0.04	0.67 ± 0.02	0.37 (7.73 × 10^−106^)
DeepSol S2	Neural network models (Khurana *et al.*, 2018)^c^. Trained and tested using a PSI: Biology dataset curated by ccSOL omics (Agostini *et al.*, 2014). Available at https://github.com/sameerkhurana10/DSOL_rv0.2.	57 (11 types)	2069.77 ± 1613.63	0.67 ± 0.02	0.23 (5.82 × 10^−41^)
DeepSol S3			2075.93 ± 1613.80	0.66 ± 0.02	0.35 (7.48 × 10^−91^)
DeepSol S1			2081.93 ± 1612.71	0.64 ± 0.03	0.39 (9.52 × 10^−116^)
CamSol intrinsic web server	Linear and logistic regression models (Sormanni *et al.*, 2015, 2017). Trained and tested using previously published datasets (Família *et al.*, 2015).Available at http://www-vendruscolo.ch.cam.ac.uk/camsolmethod.html.	4	NA	0.66 ± 0.01	0.44 (4.53 × 10^−148^)
PaRSnIP	Gradient boosting machine model (Rawi *et al.*, 2018). Trained and tested using a PSI: Biology dataset curated by ccSOL omics (Agostini *et al.*, 2014). Available at https://github.com/RedaRawi/PaRSnIP.	8477 (14 types)	2055.50 ± 1621.11	0.61 ± 0.02	0.29 (3.57 × 10^−65^)
Wilkinson–Harrison model	Linear model using charge average and turn-forming residue fraction (Wilkinson and Harrison, 1991; Davis *et al.*, 1999; Harrison, 2000). Available at https://github.com/brunoV/bio-tools-solubility-wilkinson.	2	0.09 ± 0.00	0.55 ± 0.03	–0.06 (1.16 × 10^−4^)
ccSOL omics web server	Support vector machine model (Agostini *et al.*, 2014). Trained and tested using a PSI: Biology dataset curated in-house. Available at http://s.tartaglialab.com/new_submission/ccsol_omics_file.	5	NA	0.51 ± 0.01	–0.02 (0.18)

*Note:* Boldface values are the best results.

AUC, Area Under the ROC Curve; NA, not applicable; PDB, Protein Data Bank; PSI: Biology, Protein Structure Initiative: Biology; ROC, Receiver Operating Characteristic; *R_s_*, Spearman’s rho; SWI, Solubility-Weighted Index; s, seconds.

a The wall time was reported at the level of machine precision (mean seconds ± standard deviation). A total of 10 sequences were chosen from the PSI: Biology and eSOL datasets, related to Figure 4B and Supplementary Table S7 (see Section 2).

b For SWI, mean AUC ± standard deviation was calculated from a 10-fold cross-validation (see Section 2). For other tools, no cross-validations were done as the AUC scores were calculated directly from the individual subsets used for cross-validation.

c DeepSol reports solubility prediction as probability and binary classes. The probability of solubility was used to calculate AUC and Spearman’s correlation due to better results.

To benchmark the wall time of solubility prediction tools, we selected 10 sequences that span a large range of lengths from the PSI: Biology and eSOL datasets (from 36 to 2389 residues). All the tools were run and timed using a single process without using GPUs on a high performance computer [/usr/bin/time -f ‘%E’ <command>; CentOS Linux 7 (Core) operating system, 72 cores in 2 × Broadwell nodes (E5-2695v4, 2.1 GHz, dual socket 18 cores per socket), 528 GiB memory]. Single sequence fasta files were used as input files.

### 2.4 SWI

To improve protein solubility prediction, we optimized Smith *et al.*’s normalized *B*-factors using the PSI: Biology dataset (Fig. 2). To avoid including homologous sequences in the test and training sets, we clustered the PSI: Biology targets using USEARCH v11.0.667, 32-bit (Edgar, 2010). His-tag sequences were removed from all sequences before clustering to avoid false cluster inclusions. We obtained 5050 clusters using the parameters: -cluster_fast <input_file> -id 0.4 -msaout <output_file> -threads 4. These clusters were grouped into 10 subsets with ∼1200 sequences per subset manually. The subsequent steps were carried out using sequences with His-tags.

We iteratively refined the weights of amino acid residues for solubility scoring using a 10-fold cross-validation, in which a maximized Area Under the ROC Curve (AUC) was the target (Fig. 2A). Since AUC is non-differentiable, we used the Nelder–Mead optimization method (implemented in SciPy v1.2.0), which is a derivative-free, heuristic, simplex-based optimization (Millman and Aivazis, 2011; Nelder and Mead, 1965; Oliphant, 2007). For each step in cross-validation, we used bootstrap resamplings containing 1000 soluble and 1000 insoluble proteins. Optimization was carried out for each sample, giving 1000 sets of weights. The arithmetic mean of these weights was used to determine the training and test AUC for the cross-validation step.

### 2.5 Bit score

To examine the enrichment of amino acid residues in soluble and insoluble proteins, we compute the bit scores for each residue in the PSI: Biology soluble and insoluble groups (Supplementary Fig. S7A). The count of each residue (*x*) in each group was normalized by the total number of residues in that group. We used the normalized count of amino acid residues using the eSOL *E.coli* sequences as the background. The bit score of residue *x* for soluble or insoluble group is then given by the following equation:

(5) $$\text{bit}\;\text{score}(x_{i})={\;\text{log}\;}_{2}\frac{f_{i}(x)}{f_{\text{eSOL}}(x)},i=[\text{soluble},\text{insoluble}]$$

where $f_{i}(x)$ is the normalized count of residue *x* in the PSI: Biology soluble or insoluble group and $f_{\text{eSOL}}(x)$ is the normalized count in the eSOL sequences.

For a control, random protein sequences were generated with incremental lengths, starting from a length of 50 residues to 6000 residues with a step size of 50 residues. A hundred random sequences were generated for each length, giving a total of 12 000 unique random sequences.

### 2.6 The SoDoPE web server

To estimate the probability of solubility using SWI, we fitted the following logistic regression to the PSI: Biology dataset:

(6) $$\text{probability}\;\text{of}\;\text{solubility}=\frac{1}{1+\text{exp}(-(ax+b))}$$

where *x* is the SWI of a given protein sequence, *a* = 81.05812 and b=−62.7775. The *P*-value of log-likelihood ratio test was below machine’s underflow level. Equation (6) can be used to predict the solubility of a protein sequence given that the protein is successfully expressed in *E.coli* (Supplementary Table S8).

On this basis, we developed a solubility prediction web service called SoDoPE (Soluble Domain for Protein Expression). Our web server accepts either a nucleotide or amino acid sequence. Upon sequence submission, a query is sent to the HMMER web server to annotate protein domains (https://www.ebi.ac.uk/Tools/hmmer/) (Potter *et al.*, 2018). Once the protein domains are identified, users can choose a domain or any custom region (including full-length sequence) to examine the probability of solubility, flexibility and Grand Average of Hydropathy (GRAVY). This functionality enables protein biochemists to plan their experiments and opt for the domains or regions with high probability of solubility. Furthermore, we implemented a simulated annealing algorithm that maximized the probability of solubility for a given region by generating a list of regions with extended boundaries. Users can also predict the improvement in solubility by selecting a commonly used solubility tag or a custom tag.

We linked SoDoPE with TIsigner, which is our existing web server for optimizing the accessibility of translation initiation site (Bhandari *et al.*, 2019). This pipeline allows users to predict and optimize both protein expression and solubility for a gene of interest. The SoDoPE web server is freely available at https://tisigner.com/sodope.

### 2.7 Statistical analysis

Data analysis was done using Pandas v0.25.3 (McKinney, 2010), scikit-learn v0.20.2 (Pedregosa *et al.*, 2011), numpy v1.16.2 (van der Walt *et al.*, 2011) and statsmodel v0.10.1 (Seabold and Perktold, 2010). Plots were generated using Matplotlib v3.0.2 (Hunter, 2007) and Seaborn v0.9.0 (Waskom *et al.*, 2014).

### 2.8 Code and data availability

Jupyter notebook of our analysis can be found at https://github.com/Gardner-BinfLab/SoDoPE_paper_2020. The source code for our solubility prediction server (SoDoPE) can be found at https://github.com/Gardner-BinfLab/TISIGNER-ReactJS.

## 3 Results

### 3.1 Global structural flexibility performs well at predicting protein solubility

We sought to understand what makes a protein soluble, and develop a fast and accurate approach for solubility prediction. To determine which protein sequence properties can accurately predict protein solubility, we analyzed 12 216 target proteins from over 196 species that were expressed in *E.coli* (Acton *et al.*, 2005; Chen *et al.*, 2004) (the PSI: Biology dataset; see Supplementary Fig. S1 and Table S1A). These proteins were expressed either with a C-terminal or N-terminal polyhistidine fusion tag (pET21_NESG and pET15_NESG expression vectors, *N* = 8780 and 3436, respectively). The protein entries were previously curated and classified as ‘Protein_Soluble’ or ‘Tested_Not_Soluble’ (Seiler *et al.*, 2014), based on the soluble analysis of cell lysate using SDS-PAGE (Xiao *et al.*, 2010). Both the expression system and solubility analysis method are routinely used in the labs (Costa *et al.*, 2014). This large collection of dataset captures a wide variety of protein solubility issues.

**Fig. 1.**
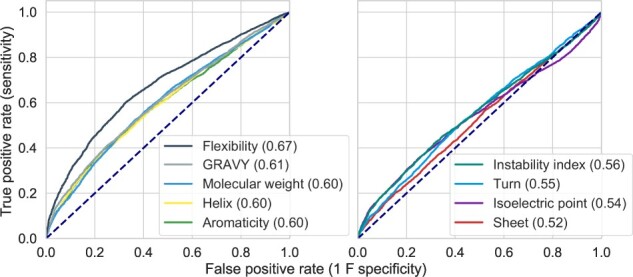
Global structural flexibility outperforms other standard protein sequence properties in protein solubility prediction. ROC analysis of the standard protein sequence features for predicting the solubility of 12 216 recombinant proteins expressed in *E.coli* (the PSI: Biology dataset). The ROC curves are shown in two separate panels for clarity. AUC scores (perfect = 1.00, random = 0.50) are shown in parentheses. Dashed lines denote the performance of random classifiers. See also Supplementary Figure S2 and Table S2. AUC, Area Under the ROC Curve; GRAVY, Grand Average of Hydropathy; PSI: Biology, Protein Structure Initiative: Biology; ROC, Receiver Operating Characteristic

We evaluated nine standard and 9920 miscellaneous protein sequence properties using the Biopython’s ProtParam module and ‘protr’ R package, respectively (Cock *et al.*, 2009; Xiao *et al.*, 2015). For example, the standard properties include the GRAVY, secondary structure propensities, protein structural flexibility, etc., whereas miscellaneous properties include amino acid composition, autocorrelation, etc. Strikingly, protein structural flexibility outperformed other features in solubility prediction (AUC = 0.67; Fig. 1, Supplementary Fig. S2 and Table S2).

### 3.2 The SWI is an improved predictor of solubility

Protein structural flexibility, in particular, the flexibility of local regions, is often associated with function (Craveur *et al.*, 2015). The local flexibility of an amino acid residue *i* can be written as:

(1) $$\begin{array}{c} f_{i}=\frac{1}{5.25}\times [B_{i}+0.8125(B_{i-1}+B_{i+1})\\ +0.625(B_{i-2}+B_{i+2})+0.4375(B_{i-3}+B_{i+3})\\ +0.25(B_{i-4}+B_{i+4})] \end{array}$$

where *B_i_* denotes the normalized *B*-factor of amino acid residue *i*. These normalized *B*-factors were previously derived from the *B*-factors extracted from protein crystal structures (Karplus and Schulz, 1985; Ragone *et al.*, 1989; Smith *et al.*, 2003; Vihinen *et al.*, 1994) (see also Supplementary Material). These normalized *B*-factors can be applied to any protein sequences without crystallographic data for flexibility prediction, e.g. as implemented in Biopython.

To predict global protein structural flexibility *F* (as in Fig. 1), *F* can be calculated as the sliding window average of normalized *B*-factors (i.e. the arithmetic mean of *f_i_*) (Cock *et al.*, 2009; Vihinen *et al.*, 1994).

(2) $$F=\langle f_{i}\rangle$$

Therefore, we can simplify Equation (1) by setting $f\prime_{i}=B_{i}$ like a zeroth-order Markov model. The simplified global flexibility F′ is then the arithmetic mean of normalized *B*-factors (see Supplementary Material for mathematical proof).

(3) $$F\prime =\langle f\prime_{i}\rangle =\langle B_{i}\rangle$$

We found a strong correlation between *F* and F′ for the PSI: Biology dataset (Spearman’s rho = 0.98, *P*-value below machine’s underflow level). Hence, the sliding window approach [Equations (1) and (2)] is not necessary for this purpose.

We applied this arithmetic mean approach (i.e. sequence composition scoring) to the PSI: Biology dataset using four sets of previously published, normalized *B*-factors (Bhaskaran and Ponnuswamy, 1988; Ragone *et al.*, 1989; Smith *et al.*, 2003; Vihinen *et al.*, 1994). Among these sets of *B*-factors, sequence composition scoring using the most recently published set of normalized *B*-factors produced the highest AUC score (AUC = 0.66; Supplementary Fig. S3).

**Fig. 2.**
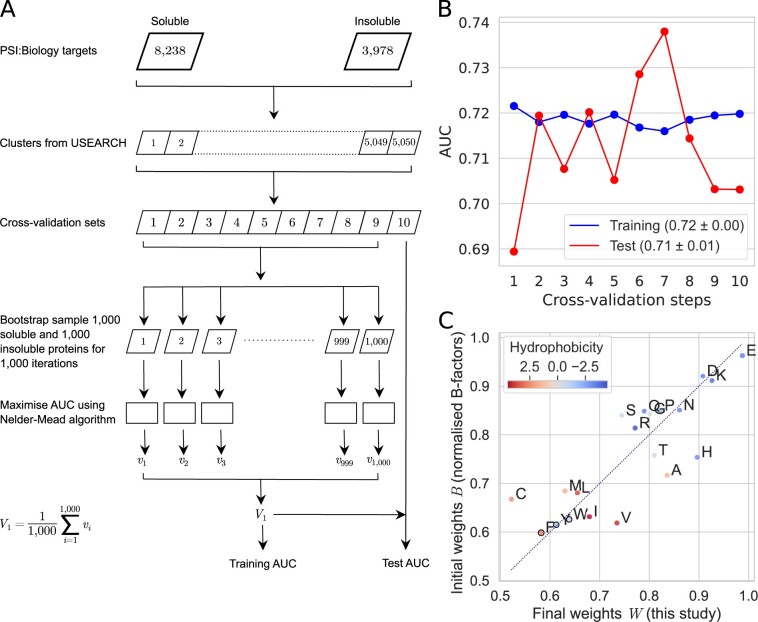
Derivation of the SWI. (**A**) Flow chart shows an iterative refinement of the weights of amino acid residues for solubility prediction. Each cross-validation step used separate sequence similarity clusters for training and testing. Furthermore, bootstrapping was used to resample each training set, avoiding training and testing on similar sequences. The solubility scores of protein sequences were calculated using a sequence composition scoring approach. These scores were used to compute the AUC scores for training and test datasets. (**B**) Training and test performance of solubility prediction using optimized weights for 20 amino acid residues in a 10-fold cross-validation (mean AUC ± standard deviation). Related data and figures are available as Supplementary Table S3 and Figures S4 and S5. (**C**) Comparison between the 20 initial and final weights for amino acid residues. The final weights $W=\langle V_{i}\rangle ,\;1\leq i\leq 10$ were used to calculate the solubility score of a protein sequence (SWI) in the four subsequent analyses. Filled circles, which represent amino acid residues, are colored by hydrophobicity (Kyte and Doolittle, 1982). Solid black circles denote aromatic residues phenylalanine (F), tyrosine (Y), tryptophan (W). Dotted diagonal line represents no change in weight. See also Supplementary Table S4. AUC, Area Under the ROC Curve; ROC, Receiver Operating Characteristic. (Color version of this figure is available at *Bioinformatics* online.)

To improve the prediction accuracy of solubility, we iteratively refined the weights of amino acid residues using the Nelder–Mead optimization algorithm (Nelder and Mead, 1965) (Fig. 2). Smith *et al.*’s normalized *B*-factors were used as initial weights. To avoid testing and training on similar sequences, we generated 10 cross-validation sets with a maximized heterogeneity between these subsets (i.e. no similar sequences between subsets). We clustered all 12 216 PSI: Biology protein sequences by a 40% similarity threshold using USEARCH to produce 5050 clusters with remote between-cluster similarity (see Section 2 and Supplementary Fig. S4). The clusters were grouped into 10 cross-validation sets of ∼1200 sequences each. As about 12% of clusters contain a mix of soluble and insoluble proteins, we avoided selecting a representative sequence for each cluster (Supplementary Fig. S4C). Furthermore, to avoid overfitting due to sequence similarity and imbalanced classes, we performed 1000 bootstrap resamplings for each cross-validation step (Fig. 2A and Supplementary Fig. S5). We calculated the solubility scores using the optimized weights and the AUC scores for each cross-validation step as shown in Figure 2A. Our training and test AUC scores were 0.72 ± 0.00 and 0.71 ± 0.01, respectively, showing a 7.5% improvement over flexibility in solubility prediction (mean ± standard deviation; Fig. 2B and Supplementary Table S3).

The final weights were derived from the arithmetic means of the weights for individual amino acid residues obtained from cross-validation (Fig. 2 and Supplementary Table S4). We observed over a 20% change on the weights for cysteine (C) and histidine (H) residues (Fig. 2C and Supplementary Table S4). These results are in agreement with the contributions of cysteine and histidine residues as shown in Supplementary Figure S2B. We call the solubility score of a protein sequence calculated using the final weights the SWI:

(4) $$\mathrm{SWI}=\langle W_{i}\rangle$$

where *W_i_* is the optimized weight of residue *i*.

To validate the cross-validation results, we used a dataset independent of the PSI: Biology known as eSOL (Niwa *et al.*, 2009) (Supplementary Table S1B). This dataset consists of the solubility percentages of *E.coli* proteins determined using an *E.coli* cell-free system (*N* = 3198). Our solubility scoring using the final weights showed a significant improvement in correlation with *E.coli* protein solubility over the initial weights (Smith *et al.*’s normalized *B*-factors) [Spearman’s rho of 0.50 (*P* = $2.51\times 10^{-205}$) versus 0.40 (*P* = $4.57\times 10^{-120}$)]. We repeated the correlation analysis by removing extra amino acid residues including His-tags from the eSOL sequences (MRGSHHHHHHTDPALRA and GLCGR at the N- and C-termini, respectively). This artificial dataset was created based on the assumption that His-tags have little effect on solubility. We observed a slight decrease in correlation for this artificial dataset (Spearman’s rho = 0.47, *P* = $3.67\times 10^{-176}$), which may be due to the effects of His-tags in solubility and/or the limitation(s) of our approach that may overfit to His-tag fusion proteins.

**Fig. 3.**
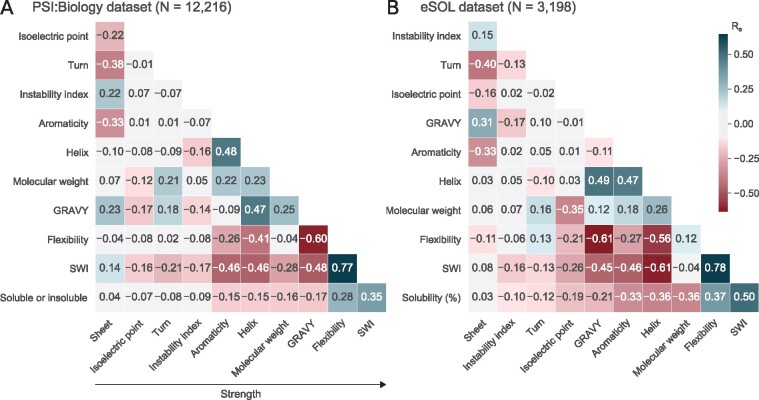
SWI strongly correlates with protein solubility. (**A**) Correlation matrix plot of the solubility of recombinant proteins expressed in *E.coli* and their standard protein sequence properties and SWI. These recombinant proteins are the PSI: Biology targets (*N* = 12 216) with a binary solubility status of ‘Protein_Soluble’ or ‘Tested_Not_Soluble’. Related data are available as Supplementary Table S5. (**B**) Correlation matrix plot of the solubility percentages of *E.coli* proteins and their standard protein sequence properties and SWI. The solubility percentages were previously determined using an *E.coli* cell-free system (eSOL, *N* = 3198). Related data are available as Supplementary Table S6. GRAVY, Grand Average of Hydropathy; PSI: Biology, Protein Structure Initiative: Biology; *R_s_*, Spearman’s rho; SWI, Solubility-Weighted Index

We performed Spearman’s correlation analysis for both the PSI: Biology and eSOL datasets. SWI shows the strongest correlation with solubility compared to the standard and 9920 miscellaneous sequence properties (Fig. 3 and Supplementary Fig. S2, respectively; see also Supplementary Tables S2B, S5 and S6). SWI strongly correlates with flexibility, suggesting that SWI is also a good proxy for global structural flexibility.

We asked whether protein solubility can be predicted by surface amino acid residues. To address this question, we examined a previously published dataset for the protein surface ‘stickiness’ of 397 *E.coli* proteins (Levy *et al.*, 2012). This dataset has the annotation for surface residues based on previously solved protein crystal structures. We observed little correlation between the protein surface ‘stickiness’ and the solubility data from eSOL (Spearman’s rho = 0.05, *P* = 0.34, *N* = 348; Supplementary Fig. S6A). Next, we evaluated if amino acid composition scoring using surface residues is sufficient, in which optimizing only the weights of surface residues should achieve similar or better results than SWI. As above, we iteratively refined the weights of surface residues using the Nelder–Mead optimization algorithm. The method was initialized with Smith *et al.*’s normalized *B*-factors and a maximized correlation coefficient was the target. However, a low correlation was obtained upon convergence (Spearman’s rho = 0.18, *P* = $7.20\times 10^{-4}$; Supplementary Fig. S6B). In contrast, the SWI of the full-length sequences has a much stronger correlation with solubility (Spearman’s rho = 0.46, *P* = $2.97\times 10^{-19}$; Supplementary Fig. S6C). These results show that the full-length of sequences contributes to protein solubility, not just surface residues, suggesting that solubility is modulated by cotranslational folding (Davis *et al.*, 1999; Natan *et al.*, 2018).

To understand the properties of soluble and insoluble proteins, we determined the enrichment of amino acid residues in the PSI: Biology targets relative to the eSOL sequences (see Section 2). We observed that the PSI: Biology targets are enriched in charged residues lysine (K), glutamate (E) and aspartate (D), and depleted in aromatic residues tryptophan (W), albeit to a lesser extend for insoluble proteins (Supplementary Fig. S7A). As expected, cysteine residues (C) are enriched in the PSI: Biology insoluble proteins, supporting previous findings that cysteine residues contribute to poor solubility in the *E.coli* expression system (Diaz *et al.*, 2010; Wilkinson and Harrison, 1991).

In addition, we compared the distributions of the SWI scores of soluble and insoluble proteins in the PSI: Biology and eSOL datasets. We included an analysis of random sequences to confirm whether SWI can distinguish between biological and random sequences. In general, the SWI scores of soluble proteins are higher than those of insoluble proteins (Supplementary Fig. S7B), and the SWI scores of true biological sequences are higher than those of random sequences, addressing our concern about the potential flaw of this position independent, sequence composition scoring approach.

### 3.3 SWI outperforms many protein solubility prediction tools

To confirm the usefulness of SWI in solubility prediction, we compared SWI with the existing tools CamSol v2.1 (Sormanni *et al.*, 2015, 2017), ccSOL omics (Agostini *et al.*, 2014), DeepSol v0.3 (Khurana *et al.*, 2018), PaRSnIP (Rawi *et al.*, 2018), Protein-Sol (Hebditch *et al.*, 2017) and the Wilkinson–Harrison model (Davis *et al.*, 1999; Harrison, 2000; Wilkinson and Harrison, 1991). We did not include the specialized tools that model protein structural information such as surface geometry, surface charges and solvent accessibility because these tools require prior knowledge of protein tertiary structure. For example, Aggrescan3D and SOLart accept only PDB files that can be either downloaded from the Protein Data Bank or produced using a homology modeling program (Hou *et al.*, 2019; Kuriata *et al.*, 2019).

**Fig. 4.**
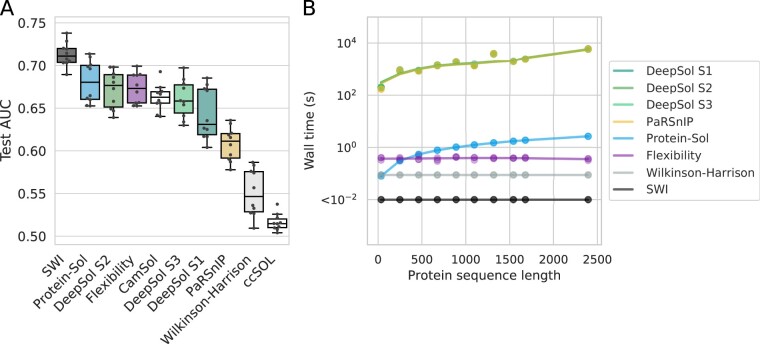
SWI outperforms existing protein solubility prediction tools. (**A**) Prediction accuracy of solubility prediction tools using the above cross-validation sets (Fig. 2A). For SWI, the test AUC scores were calculated from a 10-fold cross-validation (i.e. a boxplot representation of Fig. 2B). For other tools, no cross-validations were done as the AUC scores were calculated directly from the individual subsets used for cross-validation. CamSol and ccSOL omics are only available as web servers (no fill colors). (**B**) Wall time of protein solubility prediction tools per sequence (log scale). All command line tools were run three times using 10 sequences selected from the PSI: Biology and eSOL datasets. Related data are available as Supplementary Table S7. AUC, Area Under the ROC Curve; PSI: Biology, Protein Structure Initiative: Biology; ROC, Receiver Operating Characteristic; SWI, Solubility-Weighted Index; s, seconds. (Color version of this figure is available at *Bioinformatics* online.)

SWI outperforms other tools except for Protein-Sol in predicting *E.coli* protein solubility (Fig. 4A and Table 1). The test AUC scores of SWI were also less variable than most of the other tools, suggesting that SWI is less prone to overfitting (Figs 2A and 4A). Our SWI C program is also the fastest solubility prediction algorithm (Fig. 4B, Table 1 and Supplementary Table S7).

## 4 Discussion

The profile of normalized *B*-factors along a protein sequence can be used to infer the flexibility and dynamics of the protein structure (Karplus and Schulz, 1985; Vihinen *et al.*, 1994). Protein structural flexibility has been associated with conformal variations, functions, thermal stability, ligand binding and disordered regions (Ma, 2005; Radivojac, 2004; Schlessinger and Rost, 2005; Teague, 2003; Vihinen, 1987; Yin *et al.*, 2011; Yuan *et al.*, 2005). However, the use of flexibility in solubility prediction has been overlooked although their relationship has previously been noted (Tsumoto *et al.*, 2003). In this study, we have shown that flexibility strongly correlates with solubility (Fig. 3). Based on the normalized *B*-factors used to compute flexibility, we have derived a new position and length independent weights to score the solubility of a given protein sequence (i.e. sequence composition-based score). We call this protein solubility score as SWI.

Upon further inspection, we observe some interesting properties in SWI. SWI anti-correlates with helix propensity, GRAVY, aromaticity and isoelectric point (Figs 2C and 3), suggesting that SWI incorporates the key propensities affecting solubility. Amino acid residues with a lower aromaticity or hydrophilic are known to improve protein solubility (Han *et al.*, 2019; Kramer *et al.*, 2012; Niwa *et al.*, 2009; Trevino *et al.*, 2007; Warwicker *et al.*, 2014; Wilkinson and Harrison, 1991). Consistent with previous studies, the charged residues aspartate (D), glutamate (E) and lysine (K) are associated with high solubility, whereas the aromatic residues phenylalanine (F), tryptophan (W) and tyrosine (Y) are associated with low solubility (Fig. 2C and Supplementary Fig. S7). Cysteine residue (C) has the lowest weight, probably because disulfide bonds could not be properly formed in the *E.coli* expression hosts (Aslund and Beckwith, 1999; Jia and Jeon, 2016; Rosano and Ceccarelli, 2014; Stewart *et al.*, 1998). The weights are likely different if the solubility analysis was done using the reductase-deficient, *E.coli* Origami host strains or eukaryotic hosts.

Higher helix propensity has been reported to increase solubility (Huang *et al.*, 2012; Idicula-Thomas and Balaji, 2005). However, our analysis has shown that helical and turn propensities anti-correlate with solubility, whereas sheet propensity lacks correlation with solubility, suggesting that disordered regions may tend to be more soluble (Fig. 3). In accordance with these, SWI has stronger negative correlations with helix and turn propensities. Our findings also suggest that protein solubility can be largely explained by overall amino acid composition, not just the surface amino acid residues. This idea aligns with our understanding that protein solubility and folding are closely linked, and folding occurs cotranslationally, a complex process that is driven various intrinsic and extrinsic factors (Chiti *et al.*, 2003; Davis *et al.*, 1999; Diaz *et al.*, 2010; Natan *et al.*, 2018; Tartaglia *et al.*, 2004; Wilkinson and Harrison, 1991). However, it is unclear why sheet propensity has little contribution to solubility as *β*-sheets have been shown to link closely with protein aggregation (Idicula-Thomas and Balaji, 2005).

We conclude that SWI is a well-balanced index that is derived from a simple sequence composition scoring method. To demonstrate the usefulness of SWI, we developed a web server called SoDoPE (https://tisigner.com/sodope). SoDoPE calculates the probability of solubility of a user-selected region based on SWI, which can either be a full-length or a partial sequence (see Section 2 and Supplementary Table S8). This implementation is based on our observation that some protein domains tend to be more soluble than the others, and these soluble domains may enhance protein solubility as a whole. To demonstrate this point, we used SoDoPE to analyze three commercial monoclonal antibodies and the proteomes of the severe acute respiratory syndrome coronaviruses (SARS-CoV and SARS-CoV-2) (Marra *et al.*, 2003; Wang *et al.*, 2009; Wu *et al.*, 2020) (Supplementary Figs S8 and S9). SoDoPE also provides options for solubility prediction at the presence of solubility-enhancing tags. Similarly, these fusion tags may act as soluble ‘protein domains’ that can outweigh the aggregation propensity of insoluble proteins. However, some soluble fusion proteins may become insoluble after proteolytic cleavage of solubility tags (Lebendiker and Danieli, 2014). In addition, SoDoPE is integrated with TIsigner, a web service for optimizing protein expression (Bhandari *et al.*, 2019). This pipeline provides a holistic approach to improve the outcome of recombinant protein expression.

## Supplementary Material

btaa578_supplementary_data
